# Bone safety of dual-release hydrocortisone in patients with autoimmune primary adrenal insufficiency

**DOI:** 10.3389/fendo.2023.1234237

**Published:** 2023-09-11

**Authors:** Fabio Bioletto, Marco Barale, Mirko Parasiliti-Caprino, Jacopo Giannelli, Lorenzo Campioni, Vincenzo Cappiello, Maria Chiara Di Carlo, Ezio Ghigo, Massimo Procopio, Roberta Giordano

**Affiliations:** ^1^Division of Endocrinology, Diabetes and Metabolism, Department of Medical Sciences, University of Turin, Turin, Italy; ^2^Department of Clinical and Biological Sciences, University of Turin, Turin, Italy

**Keywords:** primary adrenal insufficiency, bone turnover markers, bone mineral density, trabecular bone score, glucocorticoid replacement therapy, dual-release hydrocortisone

## Abstract

**Background:**

Conventional glucocorticoids (C-GC) replacement regimens have a detrimental effect on skeletal health in patients with adrenal insufficiency (AI), ultimately leading to an increased fracture risk. The novel dual-release hydrocortisone (DR-HC) formulations are characterized by a more favourable safety profile on various clinical endpoints. Data comparing the impact of C-GC and DR-HC on bone, however, are scarce.

**Methods:**

Twenty-seven patients with autoimmune primary AI (PAI; 13 treated with C-GC and 14 treated with DR-HC) were evaluated to compare bone-related parameters between the two treatment groups.

**Results:**

No significant differences between the two treatments groups were observed with respect to bone turnover markers. Patients treated with C-GC showed a lower bone mineral density (BMD) at lumbar spine (LS; 0.791 ± 0.195 vs. 0.942 ± 0.124 g/cm^2^, p=0.025) and at femoral neck (FN; 0.633 ± 0.114 vs. 0.716 ± 0.088 g/cm^2^, p=0.045). Moreover, they were characterized by a lower trabecular bone score (TBS; 1.236 ± 0.035 vs. 1.383 ± 0.030, p=0.004) and by a higher mean number of vertebral fractures per patient (0.75 vs. 0 fractures, p=0.002). TBS was the best predictor of fracture risk, with a pseudo-R^2^ of 0.593; moreover, at mediation analysis, it was able to fully explain the observed detrimental effect of C-GC, compared to DR-HC, on fracture risk.

**Conclusions:**

These results suggest that DR-HC is associated with less bone-related complications compared to C-GC in patients with PAI. Moreover, TBS seems to play a pivotal role in the mediation of the relationship between glucocorticoid treatment regimens and fracture risk.

## Introduction

Patients affected by adrenal insufficiency (AI) require the chronic administration of a glucocorticoid replacement therapy (1). Although the aim of replacement therapies is to simulate as much as possible the physiological cortisol secretion (1), conventional glucocorticoid (C-GC) replacement regimens with hydrocortisone or cortisone acetate are not able to fully mimic the normal hormonal rhythm, inducing serum cortisol peaks beyond physiological levels (2, 3). This determines a mild but persistent glucocorticoid excess with a poor diurnal exposure-time profile (4, 5), which in the long-term leads to detrimental effects at different levels. Patients with AI, in fact, present an increased morbidity and mortality compared to the general population, mainly due to a worse metabolic profile and to a higher risk of cardiovascular diseases (6–9).

The exogenous administration of systemic glucocorticoids exerts a detrimental effect on bone metabolism, ultimately leading to an increased fracture risk, which is related both to the dose and to the duration of the ongoing glucocorticoid treatment (10–14). From a pathophysiological point of view, these adverse effects are mostly due to the persistent suppression of bone formation, which is determined by a direct inhibitory effect of glucocorticoids on osteoblast differentiation and function (11, 15). Nevertheless, an early but transient increase in bone resorption is also present, due to an initial stimulation of the differentiation, maturation and survival of osteoclasts (11, 15).

Even though the alteration of bone metabolism is mainly reported in case of glucocorticoid treatments for immunosuppressive and anti-inflammatory purposes, a long-term replacement therapy with C-GC regimens has been demonstrated to exert a negative impact on skeletal health as well, though to a lesser extent (16, 17). A decrease in bone mineral density (BMD) and an increase in fracture risk have been reported in adult patients receiving C-GC replacement therapy for either primary adrenal insufficiency (PAI), secondary adrenal insufficiency (SAI) or congenital adrenal hyperplasia (CAH) (16–18); moreover, a correlation between these outcomes and the cumulative glucocorticoid dose has been observed (19, 20).

In recent years, a dual-release hydrocortisone (DR-HC) preparation (Plenadren^®^) was developed to maintain cortisol levels in a more physiological range, better reproducing the endogenous cortisol rhythm both when evaluated by serum cortisol (21, 22) and by salivary cortisol (23) levels. Compared to C-GC, this formulation has been demonstrated to have a more favourable clinical profile, with better outcomes with respect to blood pressure control, glucose metabolism, lipid metabolism, body weight, and health-related quality of life (22, 24–27).

Data about the impact of DR-HC on bone metabolism, however, are scarce. In a recent study by Frara et al. (28), performed on patients with SAI, a shift from C-GC to DR-HC was associated with a significant increase in BMD values at lumbar spine and femoral neck. Similar results were demonstrated by Guarnotta et al. (29) in a cohort of patients with PAI, in which a treatment with DR-HC was associated with a more favourable clinical profile in terms of BMD, bone turnover markers and, possibly, fracture risk.

Further data are thus required to better establish the safety profile of DR-HC, compared to C-GC, on skeletal health. Based on this background, we designed a clinical study aimed at comparing bone-related parameters between patients with PAI treated either with DR-HC or with C-GC replacement therapies. Both biochemical and imaging parameters were assessed, and possible mediators of the relationship between glucocorticoid treatment regimens and fracture risk were analysed.

## Methods

### Patient selection

Twenty-seven patients with PAI of autoimmune aetiology were evaluated between September 2020 and August 2021. Twenty-one were affected by autoimmune polyendocrine syndrome (APS) type 2, one by APS type 4, and five by autoimmune isolated Addison’s disease.

The following exclusion criteria were applied: (a) APS type 1; (b) hypo- or hyperparathyroidism; (c) hyperthyroidism; (d) end-stage chronic kidney disease; (e) current or previous treatment with anti-osteoporotic drugs (apart from calcium and vitamin D supplementation).

All pre-menopausal women were studied in the early follicular phase. Female patients with primary ovarian insufficiency were under appropriate replacement therapy at the time of the study, as well as male patients with primary hypogonadism. Patients with autoimmune hypothyroidism were under appropriate treatment with levothyroxine at the time of the study. Patients affected by vitamin D insufficiency received adequate supplementation.

The following parameters were evaluated for each patient: age, sex, disease duration, current treatment duration (defined as the duration of therapy with the current formulation and dosage), hydrocortisone equivalent (HCeq) dose, smoking habit, weight, body mass index (BMI), creatinine, fasting glucose, HbA1c, serum and urinary calcium, serum and urinary phosphate, parathyroid hormone (PTH), 25-OH vitamin D (25(OH)D), bone alkaline phosphatase (BAP), osteocalcin, urinary crosslinks, bone mineral density (BMD) at lumbar spine (LS), femoral neck (FN) and total hip (TH), trabecular bone score (TBS) at LS, and number of vertebral fractures at vertebral morphometry.

The study was approved by the local Ethics Committee and was in accordance with the principles of the Declaration of Helsinki. Written informed consent was obtained from all included patients.

### Analytical methods

Blood samples were taken in the morning between 8 and 9 am, after an overnight fast. Serum PTH (ng/L), BAP (µg/L) and osteocalcin (μg/L) were tested by chemiluminescence immunoassay (CLIA; Liason Analyzer, DiaSorin, Saluggia, Italy). Serum 25(OH)D (µg/L) was tested by chemiluminescence microparticle immunoassay (CMIA; Alinity system, Abbott Laboratories, Chicago, Illinois, USA). Urinary crosslinks (nmol/mmol of creatinine) were tested by crosslinks method (Chromsystems, Grafelfings, Germany) with fluorescent detection based on high performance liquid chromatography (HPLC; Prominence System, Shimadzu Corporation, Kyoto, Japan). All other biochemical variables were assayed in plasma, serum or urine using standard methods.

### BMD, TBS and vertebral morphometry analysis

BMD was measured by dual energy x-ray absorptiometry (DXA) at L1-4 LS, FN and TH, using a Hologic QDR 4500 densitometer. Fractured vertebrae were excluded from the calculation of BMD and its derived parameters. The coefficient of variation of BMD measurements was equal to 1.0% at all examined sites. T-scores were calculated by comparing BMD results with those obtained in a sex-matched Caucasian population at peak of bone mass. Z-scores were calculated by comparing BMD results with those obtained in an age and sex-matched Caucasian population. TBS was evaluated from LS DXA scans using the TBS iNsight^®^ software (version 3.0.2.0, Medimaps SASU, Pessac, France). The coefficient of variation of TBS measurements was equal to 1.2%. Vertebral morphometry was performed on lateral spine images obtained by DXA scan; conventional spinal radiographs (T4-L4) in the lateral and the anteroposterior projections would have been performed in case on unclear or inconclusive findings at lateral spine DXA imaging (30), but this was not necessary for any patient in the present study.

### Statistical analysis

Continuous data were summarised using mean and standard deviation (SD) for normally distributed variables, and median and interquartile range (IQR) for non-normally distributed variables. Count data were summarised using the mean number of observed events per patient. Categorical data were summarised using percent values. Normality of continuous variables was assessed through Kolmogorov-Smirnov test. Differences between treatment groups were evaluated by Student t-test or by Mann-Whitney U-test for continuous variables, by exact Poisson regression for count variables, and by Fisher’s exact test for categorical variables. The same tests have been applied to evaluate differences between patients with and without vertebral fractures.

All the bone-related parameters that were significantly different between treatment groups at univariate analysis were further analysed by analysis of covariance (ANCOVA) if continuous-type, or by multivariable exact Poisson regression if count-type; adjustments for age, disease duration, current treatment duration, HCeq dose, 25(OH)D levels, and HbA1c levels were performed.

Univariate and multivariable exact Poisson regressions were used to further define the most relevant factors associated with vertebral fractures, considering as potential predictors the glucocorticoid treatment regimen (C-GC vs. DR-HC), together with all the other parameters found to be significantly associated with fractures at univariate analyses. Finally, a mediation analysis was performed in order to assess whether the impact of glucocorticoid treatment regimen on fracture risk could be explained by one or more of the bone-related parameters that have been analyzed; a mediation model aims to uncover and elucidate the mechanism that lies beneath an observed relationship between an independent variable and a dependent variable via the inclusion of a third variable, known as a mediator variable (31, 32); through this approach, the total exposure-outcome effect is decomposed into direct and indirect effects, the first one being the direct exposure-outcome effect, while the second being the combination of exposure-mediator and mediator-outcome effects, as visually represented by direct acyclic graphs (31, 32); with regard to this study, thus, the total effect of glucocorticoid treatment regimen on fracture risk was decomposed to assess whether this relationship was significantly mediated by other measured variables.

A cut-off of 0.05 was adopted for the definition of statistical significance. Statistical analysis was performed using STATA 17 (StataCorp, College Station, Texas, USA).

## Results

### General characteristics of the study population

Thirteen patients were treated with C-GC replacement therapy (4 patients with cortisone acetate, divided in two or three doses; 9 patients with hydrocortisone, divided in two or three doses). Fourteen patients were treated with DR-HC (single dose in the morning). In addition, all patients were treated with fludrocortisone (0.025-0.1 mg/day in the morning). All patients reported adequate adherence to prescribed treatments. The main anthropometric, clinical and biochemical characteristics of the patients are reported in **Table 1**.

**Table 1:** Clinical characteristics of patients treated with C-GC therapy versus patients treated with DR-HC formulation.

Parameter	C-GC therapy (n = 13)	DR-HC therapy (n = 14)	p-value
Age (years)	59.5 ± 11.4	49.8 ± 15.0	0.070
Male sex (n, %)	3 (23.1)	5 (35.7)	0.678
Disease etiology/subtype			1.000
Isolated AD (n, %)	2 (15.4)	3 (21.4)	
APS type 2 (n, %)	11 (84.6)	10 (71.4)	
APS type 4 (n, %)	0 (0.0)	1 (7.1)	
Disease duration (years)	15.5 ± 12.9	17.8 ± 9.4	0.631
Current treatment duration (years) ^a^	5.0 [3.6-8.4] ^a^	7.0 [4.0-8.0] ^a^	1.000 ^a^
HCeq dose (mg/day)	20.0 ± 3.7	22.1 ± 3.8	0.149
Current smoking (n, %)	0 (0.0)	0 (0.0)	NA
Weight (kg)	62.6 ± 11.9	65.7 ± 10.0	0.483
BMI (kg/m^2^)	24.3 ± 4.3	23.6 ± 2.7	0.637
Creatinine (mg/dL)	0.88 ± 0.20	0.78 ± 0.21	0.225
Glucose (mg/dL)	90.8 ± 31.7	79.4 ± 17.3	0.252
HbA1c (mmol/mol)	46.1 ± 13.0	35.3 ± 7.1	**0.012**
Calcium (mmol/L)	2.37 ± 0.12	2.38 ± 0.30	0.932
Urinary calcium (mmol/24h)	3.16 ± 1.60	3.54 ± 2.11	0.606
Phosphate (mmol/L)	1.15 ± 0.22	1.17 ± 0.16	0.740
Urinary phosphate (mmol/24h)	19.00 ± 8.40	20.61 ± 10.21	0.660
PTH (ng/L)	27.9 ± 7.2	24.5 ± 8.7	0.270
25(OH)D (μg/L)	39.0 ± 8.1	33.3 ± 11.4	0.151
BAP (μg/L)	12.4 ± 4.1	10.8 ± 2.6	0.255
Osteocalcin (μg/L)	21.9 ± 3.2	25.5 ± 6.5	0.083
Urinary crosslinks (nmol/mmol of Cr)	26.6 ± 9.1	29.8 ± 17.1	0.556
Lumbar spine T-score	-2.01 ± 1.51	-1.10 ± 1.04	0.080
Total hip T-score	-1.47 ± 0.89	-1.26 ± 0.62	0.489
Femoral neck T-score	-1.85 ± 1.02	-1.39 ± 0.67	0.167
Lumbar spine Z-score	-1.13 ± 1.20	-0.44 ± 0.97	0.122
Total hip Z-score	-0.86 ± 0.77	-0.70 ± 0.70	0.586
Femoral neck Z-score	-1.10 ± 0.50	-0.65 ± 0.78	0.099
Lumbar spine BMD (g/cm^2^)	0.791 ± 0.195	0.942 ± 0.124	**0.025**
Total hip BMD (g/cm^2^)	0.752 ± 0.128	0.820 ± 0.091	0.128
Femoral neck BMD (g/cm^2^)	0.633 ± 0.114	0.716 ± 0.088	**0.045**
Trabecular bone score	1.236 ± 0.035	1.383 ± 0.030	**0.004**
Prevalence of vertebral fractures (%) ^b^	25.0	0	0.085
Mean n. of fractures per patient ^b,c^	0.75 ^c^	0 ^c^	**0.002** ^c^

^a^Data reported as median [IQR] and tested for differences with Mann-Whitney U-test due to the non-normal distribution of the considered parameter according to Kolmogorov-Smirnov test; ^b^ Missing data in one patient treated with conventional glucocorticoid therapy; ^c^ Count-type data tested for differences by exact Poisson regression.

25(OH)D, 25-OH vitamin D; AD, Addison’s disease; APS, autoimmune polyendocrine syndrome; BAP, bone alkaline phosphatase; BMD, bone mineral density; BMI, body mass index; C-GC, conventional glucocorticoid; Cr, creatinine; DR-HC, dual-release hydrocortisone; eGFR, estimated glomerular filtration rate; HCeq, hydrocortisone equivalent; n, number; NA, not applicable; PTH, parathyroid hormone.

Bold values indicate statistically significant results.

Comparing patients treated with C-GC and those treated with DR-HC, those belonging to the first group showed a tendency towards an older age (59.5 ± 11.4 vs. 49.8 ± 15.0 years, p=0.070), while no differences were found in terms of sex (23.1 vs. 35.7% of males, p=0.678), disease etiology/subtype (isolated PAI or APS, p=1.000), disease duration (15.5 ± 12.9 vs. 17.8 ± 9.4 years, p=0.631), current treatment duration (5.0 [IQR: 3.6-8.4] vs. 7.0 [IQR 4.0-8.0] years, p=1.000), and HCeq dose (20.0 ± 3.7 vs. 22.1 ± 3.8 mg/day, p=0.149) (**Table 1**). Weight, BMI and fasting glucose were comparable between groups, while HbA1c was higher in patients treated with C-GC than in those treated with DR-HC (46.1 ± 13.0 vs. 35.3 ± 7.1 mmol/mol, p=0.012) (**Table 1**).

### Comparison of bone-related parameters between C-GC and DR-HC replacement therapies

With respect to bone-related biochemical parameters, no differences could be observed between patients treated with C-GC and those treated with DR-HC. In particular, the two treatment groups presented with comparable levels of serum and urinary calcium, serum and urinary phosphate, 25(OH)D, and PTH (**Table 1**). A borderline-significand trend towards lower osteocalcin levels in the C-GC group could be observed (21.9 ± 3.2 vs. 25.5 ± 6.5 μg/L, p=0.083); the other evaluated bone turnover markers (i.e., BAP and urinary crosslinks) were similar between the two groups (**Table 1**).

Considering DXA evaluation, patients treated with C-GC showed a lower BMD at LS (0.791 ± 0.195 vs. 0.942 ± 0.124 g/cm^2^, p=0.025) and at FN (0.633 ± 0.114 vs. 0.716 ± 0.088 g/cm^2^, p=0.045), while only a non-significant trend could be observed at TH (0.752 ± 0.128 vs. 0.820 ± 0.091 g/cm^2^, p=0.128) (**Table 1**). At TBS analysis, lower values were observed in patients treated with C-CG compared to those treated with DR-HC (1.236 ± 0.035 vs. 1.383 ± 0.030, p=0.004) (**Table 1**). Vertebral fractures were observed in 3 patients treated with C-GC, with a total number of 9 fractures (one patient with 2 fractures, one with 3 fractures, one with 4 fractures); conversely, no vertebral fractures were observed in patients treated with DR-HC (p=0.002 for difference between groups) (**Table 1**).

All parameters found to be significant at univariate analysis were further evaluated by taking into account the main possible confounding factors (i.e., age, disease duration, current treatment duration, HCeq dose, 25(OH)D level and HbA1c), using ANCOVA for continuous-type data and multivariable exact Poisson regression for count-type data (**Table 2**). The difference between groups in LS-BMD remained statistically significant after accounting for almost all confounders (p=0.045 adjusted for age; p=0.024 adjusted for disease duration; p=0.047 adjusted for HCeq dose; p=0.047 adjusted for 25(OH)D; p=0.008 adjusted for HbA1c), with the exception of current treatment duration, for which a borderline significance was nevertheless observed (p=0.052) (**Table 2**). The difference between groups in FN-BMD remained statistically significant when accounting for disease duration (p=0.021) and HbA1c (p=0.032), but the significance was lost when adjusting the analysis for age (p=0.108), current treatment duration (p=0.135), HCeq dose (p=0.082), and 25(OH)D (p=0.107) (**Table 2**). The difference between groups in TBS remained statistically significant in all adjusted analyses (p=0.023 adjusted for age; p=0.007 adjusted for disease duration; p=0.020 adjusted for current treatment duration; p=0.007 adjusted for HCeq dose; p=0.011 adjusted for 25(OH)D; p=0.006 adjusted for HbA1c) (**Table 2**). The same held true for the difference in the mean number of fractures per patient (p=0.029 adjusted for age; p=0.013 adjusted for disease duration; p=0.004 adjusted for current treatment duration; p=0.004 adjusted for HCeq dose; p=0.008 adjusted for 25(OH)D; p=0.002 adjusted for HbA1c) (**Table 2**). Further analyses accounting for multiple confounders at the same time were not feasible due to the limited sample size.

**Table 2:** Adjusted comparison between treatment groups of bone-related parameters that were significantly different at univariate analysis.

Dependent variable	Crude p-value for difference between treatment groups	Adjusted p-value for difference between treatment groups					
		Adjustment for age	Adjustment for disease duration	Adjustment for current treatment duration	Adjustment for HCeq dose	Adjustment for 25(OH)D levels	Adjustment for HbA1c levels
Lumbar spine BMD (g/cm^2^)	0.025	0.045	0.024	0.052	0.047	0.047	0.008
Femoral neck BMD (g/cm^2^)	0.045	0.108	0.021	0.135	0.082	0.107	0.032
Trabecular bone score	0.004	0.023	0.007	0.020	0.007	0.011	0.006
Mean n. of fractures per patient	0.002	0.029	0.013	0.004	0.004	0.008	0.002

25(OH)D, 25-OH vitamin D; BMD, bone mineral density; n, number.

### Prediction of fragility fractures

Regardless of the treatment, patients with vertebral fractures were characterized by a significantly longer disease duration (30.7 ± 10.1 vs. 15.0 ± 10.2 years, p=0.021), lower LS T-score (-3.20 ± 1.92 vs. -1.32 ± 1.18, p=0.022), lower FN T-score (-2.73 ± 0.12 vs. -1.49 ± 0.83, p=0.018), lower FN-BMD (0.545 ± 0.013 vs. 0.695 ± 0.102 g/cm^2^, p=0.019), and lower TBS (1.098 ± 0.096 vs. 1.343 ± 0.117, p=0.002) compared to those without vertebral fractures (**Table 3**).

**Table 3:** Comparison of the clinical characteristics of patients with and without vertebral fractures, irrespective of the ongoing glucocorticoid treatment regimen.

Parameter	Patients without vertebral fractures (n = 23)	Patients with vertebral fractures (n = 3)	p-value
Age (years)	52.7 ± 14.3	67.7 ± 4.2	0.087
Male sex (n, %)	7 (30.4)	1 (33.3)	1.000
Disease etiology/subtype			0.562
Isolated AD (n, %)	4 (17.4)	1 (33.3)	
APS type 2 (n, %)	18 (78.3)	2 (66.7)	
APS type 4 (n, %)	1 (4.3)	0 (0.0)	
Disease duration (years)	15.0 ± 10.2	30.7 ± 10.1	**0.021**
Current treatment duration (years) ^a^	6.0 [3.4-8.0] ^a^	9.7 [3.0-32.0] ^a^	0.322
HCeq dose (mg/day)	21.6 ± 3.7	20.0 ± 0.0	0.456
Current smoking (n, %)	0 (0.0)	0 (0.0)	NA
Weight (kg)	64.6 ± 11.1	64.0 ± 11.5	0.927
BMI (kg/m^2^)	24.0 ± 3.4	24.4 ± 5.4	0.845
Creatinine (mg/dL)	0.80 ± 0.18	1.00 ± 0.34	0.115
Glucose (mg/dL)	84.9 ± 26.6	85.0 ± 24.9	0.994
HbA1c (mmol/mol)	40.3 ± 11.4	44.0 ± 16.5	0.614
Calcium (mmol/L)	2.39 ± 0.24	2.26 ± 0.09	0.341
Urinary calcium (mmol/24h)	3.43 ± 1.76	2.06 ± 2.41	0.233
Phosphate (mmol/L)	1.19 ± 0.18	1.01 ± 0.22	0.119
Urinary phosphate (mmol/24h)	19.64 ± 8.78	20.17 ± 15.98	0.929
PTH (ng/L)	25.2 ± 7.6	33.9 ± 10.1	0.083
25(OH)D (μg/L)	35.4 ± 10.6	41.4 ± 8.8	0.353
BAP (μg/L)	11.6 ± 3.6	12.3 ± 2.2	0.740
Osteocalcin (μg/L)	24.3 ± 5.6	19.5 ± 3.1	0.160
Urinary crosslinks (nmol/mmol of Cr)	29.8 ± 14.1	20.8 ± 6.2	0.293
Lumbar spine T-score	-1.32 ± 1.18	-3.20 ± 1.92	**0.022**
Total hip T-score	-1.32 ± 0.75	-1.93 ± 0.57	0.190
Femoral neck T-score	-1.49 ± 0.83	-2.73 ± 0.12	**0.018**
Lumbar spine Z-score	-0.62 ± 0.98	-1.80 ± 1.77	0.085
Total hip Z-score	-0.75 ± 0.76	-0.97 ± 0.25	0.629
Femoral neck Z-score	-0.80 ± 0.72	-1.27 ± 0.25	0.285
Lumbar spine BMD (g/cm^2^)	0.889 ± 0.168	0.740 ± 0.212	0.170
Total hip BMD (g/cm^2^)	0.802 ± 0.113	0.688 ± 0.039	0.101
Femoral neck BMD (g/cm^2^)	0.695 ± 0.102	0.545 ± 0.013	**0.019**
Trabecular bone score	1.343 ± 0.117	1.098 ± 0.096	**0.002**

^a^Data reported as median [IQR] and tested for differences with Mann-Whitney U-test due to the non-normal distribution of the considered parameter according to Kolmogorov-Smirnov test

25(OH)D, 25-OH vitamin D; AD, Addison’s disease; APS, autoimmune polyendocrine syndrome; BAP, bone alkaline phosphatase; BMD, bone mineral density; BMI, body mass index; n, number; Cr, creatinine; eGFR, estimated glomerular filtration rate; HCeq, hydrocortisone equivalent; n, number; NA, not applicable; PTH, parathyroid hormone.

Bold values indicate statistically significant results.

Among the parameters that significantly differed between patients with and without vertebral fractures, TBS was the one that showed the best predictive performance at univariate Poisson regression, with a pseudo-R^2^ of 0.593. The performance of TBS in fracture prediction could not be improved by adding to the model any of the other variables that were significant at univariate analysis, which all lost significance when adjusted for TBS values (p=0.667 for disease duration; p=0.625 for LS T-score; p=0.625 for FN T-score; p=0.625 for FN-BMD), while TBS always maintained a significant association with the outcome (p<0.01 in all models).

**Figure 1:**
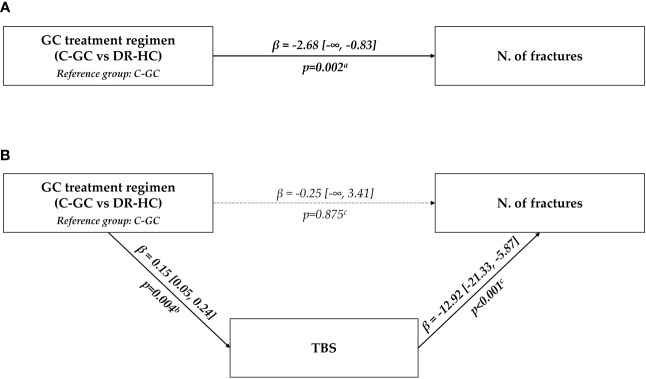
Graphical representation of the association between glucocorticoid treatment regimen (C-GC vs. DR-HC) and fracture risk. The upper panel **(A)** shows the results of the univariate analysis; the lower panel **(B)** shows the results of the multivariable mediation analysis, considering TBS as a mediating path. ^a^Univariate exact Poisson regression; ^b^Linear regression; ^c^Multivariable exact Poisson regression. C-GC, conventional glucocorticoid; DR-HC, dual-release hydrocortisone; GC, glucocorticoid; N, number; TBS, trabecular bone score.

Finally, the hypothesis of a mediating effect of TBS in the relationship between glucocorticoid treatment regimen (C-GC vs. DR-HC) and fracture risk was tested in a mediation analysis model. The retrieved results showed that, in our cohort, the detrimental effect of C-GC on fracture risk was entirely explicable through the mediation of a reduced TBS; in fact, the direct path from the treatment regimen to fracture risk lost any statistical significance (p=0.875); on the other hand, the indirect path passing through TBS showed a significant mediating effect of this variable (p=0.004 for the association between glucocorticoid treatment regimen and TBS; p<0.001 for the association between TBS and vertebral fractures) (**Figure 1**).

## Discussion

In this study, we compared the impact of two different glucocorticoid replacement regimens (C-GC vs. DR-HC) on skeletal health in a group of patients with PAI. Both biochemical and imaging parameters were assessed, and possible mediators of the relationship between glucocorticoid treatment regimens and fracture risk were analysed. Overall, our data seemed to suggest a more favorable bone-safety profile in patients treated with DR-HC; in fact, these patients displayed higher BMD values, higher TBS values, and a lower fracture rate compared to those treated with C-GC. Given the observational nature of this study, a possible role of confounding variables cannot be excluded in this regard; although not significantly, patients treated with C-GC were older than those treated with DR-HC, and this may have contributed to the differences in bone health parameters between the two groups; overall, however, our findings were essentially confirmed also after adjusting for age, disease duration, current treatment duration, HCeq dose, 25(OH)D levels and HbA1c.

To the best of our knowledge, only two studies provided, so far, a comparison between C-GC and DR-HC with respect to bone-related endpoints. The first one, published in 2018 by Frara et al. (28), was a retrospective cohort study which enrolled 14 patients with SAI who were shifted from C-GC to DR-HC upon clinical decision; this therapeutic change was associated with a significant increase in LS-BMD and FN-BMD after a follow-up of 24 months; no data on other parameters, such as bone turnover markers, TBS or fractures, were available. The second one, published in 2022 by Guarnotta et al. (29), was a retrospective cohort study which enrolled 70 patients with PAI, half of which were shifted from C-GC to DR-HC upon clinical decision; after a follow-up of 60 months, patients who switched to DR-HC displayed a significant increase in BAP, LS-BMD and FN-BMD; on the other hand, patients who continued on C-GC displayed a significant decrease in BAP, osteocalcin and LS-BMD, together with an increase in vertebral fracture rate. Overall, these results are in line with those observed in our study; however, some elements of novelty of our research should be underlined.

From a pathophysiological point of view, glucocorticoid-induced osteoporosis (GIO) is characterised by a preferential loss of trabecular bone, with a lesser impact on cortical bone; as a consequence, the spinal column is the skeletal site that is most affected, with a preferential loss of BMD at LS and a more prominent increase in vertebral fracture risk (33, 34). Overall, however, the increase in fracture risk in patients treated with chronic systemic glucocorticoids is only partially related to the observed changes in BMD (35–37). Although a reduction in BMD is present, in fact, this parameter alone has a poor discriminatory capacity in predicting fracture risk in the setting of GIO (35–37). The mechanisms underlying this BMD-independent increase in fracture risk are related to a glucocorticoid-induced impairment of bone microarchitecture, with a greater decrease in bone quality rather than in bone density (38–40).

In recent years, alternative parameters of skeletal fragility have been sought, in place of BMD, for a better fracture risk assessment in patients with primary and secondary osteoporosis (41–47), including GIO (38, 39, 48). Among these, TBS has emerged as a novel index of bone micro-architectural health and skeletal fragility (41, 42). TBS is a qualitative index that captures the mean rate of gray-level variations in LS DXA images; higher TBS values reflect a better trabecular bone microstructure, while lower TBS values indicate a trabecular microstructure impairment (41, 42). Compared to BMD, TBS is able to better characterize bone fragility in GIO, demonstrating a higher discriminatory capacity in the estimation of fracture risk (49, 50). Within the setting of AI, however, data are scarce; to our knowledge, only one study has to date evaluated TBS values in patients with AI (51); in this study, TBS was negatively correlated with age and disease duration, but no data were available comparing TBS between patients treated with C-GC and DR-HC replacement therapies, nor about its possible role as a predictor of fracture risk (51).

In our study, in agreement with previous literature (28, 29), we observed that DR-HC was associated with higher BMD values compared to C-GC, both at LS and FN. Our study, however, was the first to evaluate the possible role of TBS in this setting, with promising results. The evaluation of TBS represented a key point for a finer assessment of skeletal fragility in the enrolled cohort. In fact, the difference between treatment groups in terms of TBS was stronger, and TBS was the only DXA-derived parameter that strictly remained statistically significant in all adjustments performed by ANCOVA.

The key role of TBS in our study, however, became even more evident in the analysis of the predictors of vertebral fractures. Overall, our data showed a significant difference in terms of vertebral fracture risk between the two treatment groups, with a significantly higher number of fractures in patients treated with C-GC. This result is in line with the one reported by Guarnotta et al. (29), who reported an increase in vertebral fracture rate in patients treated with C-GC but not in those treated with DR-HC. When evaluating the possible predictors of vertebral fractures, TBS was the one that displayed the strongest association with the outcome, with a pseudo-R^2^ of 0.593. Notably, none of the other considered variables could improve this performance when added to the model, thus emphasizing the pivotal role of TBS for the assessment of fracture risk in this clinical setting.

Moreover, a mediation analysis model was used to analyze the possible mediating role of TBS in the association between glucocorticoid treatment regimens (C-GC vs. DR-HC) and fracture risk. In this analysis, the total effect of the glucocorticoid treatment regimen on fracture risk was partitioned into direct and indirect effects, considering TBS as a possible mediator. Interestingly, the whole detrimental role of C-GC on fracture risk could be explained by the indirect path passing through TBS, while the direct path from the treatment regimen to fracture risk lost any statistical significance. In other words, TBS was able to capture the entire association between the glucocorticoid treatment regimen (C-GC vs. DR-HC) and fracture risk: the treatment regimen had a significant impact on TBS, and, in turn, TBS had a significant impact on fracture rate, but no significant direct (i.e., not TBS-mediated) impact of the treatment regimen on fractures could be detected.

With respect to biochemical parameters, in our study, bone turnover markers did not significantly differ between the two treatment groups. Nevertheless, a borderline-significant trend towards lower levels of osteocalcin in patients treated with C-GC could be seen. This difference is in line with the findings by previous authors (22, 29), and suggests a stronger inhibitory effect on bone formation exerted by C-GC compared to DR-HC.

Our study had some limitations. First, it was a proof-of-concept study with a limited sample size; the obtained results should be considered as preliminary and need to be confirmed in larger cohorts. Second, it had a cross-sectional design, and did not evaluate the effect of treatments over time; this permits to infer associations, but it does not allow to establish a link of causality. Third, the assignment to treatment regimen was not randomized and, thus, even if we evaluated the influence of possible covariates by multivariable analyses, the presence of a residual confounding cannot be excluded.

In conclusion, our study suggests a better safety profile of DR-HC, compared to C-GC, in terms of bone-related complications in patients with PAI. These results are in line with the more favorable pharmacological profile of DR-HC, which is able to better reproduce the endogenous cortisol rhythm, thus leading to less side effects related to glucocorticoid excess. Notably, TBS seems to play a pivotal role in the mediation of the relationship between glucocorticoid treatment regimens and fracture risk. This finding is coherent with the specific impairment of bone microarchitecture that is determined by glucocorticoid excess, which affects bone quality rather than bone density.

## Data availability statement

The raw data supporting the conclusions of this article will be made available by the authors, without undue reservation.

## Ethics statement

The studies involving humans were approved by “Comitato Etico Interaziendale - AOU Città della Salute e della Scienza di Torino”. The studies were conducted in accordance with the local legislation and institutional requirements. The participants provided their written informed consent to participate in this study.

## Author contributions

FB contributed to data analysis and manuscript writing. MB and MP-C contributed to manuscript review and editing. JG, LC, and MCDC contributed to data collection. VC contributed to data collection and manuscript writing. EG and MP supervised the manuscript drafting. RG contributed to work conceptualization and final draft supervision. All authors approved the manuscript in its final form. All authors contributed to the article.
